# Targeting the hsa-miR-155-5p–BACH1–MMP-9 Signaling Hub in Lung Cancer: A Novel Anticancer Mechanism of Thymoquinone

**DOI:** 10.3390/biom16070955

**Published:** 2026-06-27

**Authors:** Yusuf Saleem Khan, Aisha Farhana, Alfatih Mohamed Ahmed Alnajib, Azharuddin Sajid Syed Khaja, Hatim Adam Nagi, Tarig Ginawi, Abuzar Abdulwahab Osman, Ayman Ali Mohammed Alameen, Emad Manni, Zafar Rasheed

**Affiliations:** 1 Department of Anatomy, College of Medicine, University of Ha’il, Ha’il 55476, Saudi Arabia; 2 Department of Clinical Laboratory Sciences, College of Applied Medical Sciences, Jouf University, Sakaka 72388, Saudi Arabia; aaalameen@ju.edu.sa (A.A.M.A.); emena@ju.edu.sa (E.M.); 3 Department of Surgery, College of Medicine, University of Ha’il, Ha’il 55476, Saudi Arabia; a.alnajib@uoh.edu.sa; 4 Department of Pathology, College of Medicine, University of Ha’il, Ha’il 55476, Saudi Arabia; skazharuddin@uoh.edu.sa; 5 Department of Family and Community Medicine, College of Medicine, University of Ha’il, Ha’il 55476, Saudi Arabia; ha.adam@uoh.edu.sa; 6 Department of Biochemistry, College of Medicine, University of Ha’il, Ha’il 55476, Saudi Arabia; tariginawi@gmail.com; 7 Department of Pharmacology, College of Medicine, University of Ha’il, Ha’il 55476, Saudi Arabia; a.osman@uoh.edu.sa; 8 LTT-LabTech Training Centre, 28 Russell St, South Brisbane, QLD 4101, Australia; zafar.rasheed@ltt.edu.au

**Keywords:** lung neoplasm, microRNA-155, thymoquinone, BACH1, MMP-9, neoplasm metastasis, inflammation mediators, natural compounds in cancer therapy

## Abstract

Objective: Lung cancer (LC) remains a leading cause of cancer mortality worldwide. Thymoquinone (TQ), a bioactive compound derived from *Nigella sativa*, possesses anti-inflammatory and antioxidant properties, but its precise mechanisms concerning miRNA regulation in LC are poorly defined. This study investigates the anti-cancer potential of TQ through modulation of microRNA signaling in LC. Methods: We employed an integrated approach combining bioinformatic predictions with rigorous experimental validation in A549 lung adenocarcinoma cells and SHP-77 human small-cell lung carcinoma (SCLC) cells. Bioinformatic analyses predicted miRNA targets, and experimental techniques included dual-luciferase reporter assays, miRNA inhibition, TaqMan RT-qPCR, cell-based ELISA, and Western blotting to dissect the molecular pathway. Results: We identified the transcription factor BACH1 as a direct and novel target of hsa-miR-155-5p. TQ potently suppressed interferon-γ-induced expression of both hsa-miR-155-5p and its target, BACH1. This TQ-mediated suppression led to subsequent downregulation of the key metastasis-promoter Matrix Metalloproteinase-9 (MMP-9). Genetic inhibition of miR-155-5p or direct BACH1 inhibition phenocopied the effects of TQ, confirming the functional significance of this axis. Thus, we define a novel oncogenic signaling cascade—the hsa-miR-155-5p/BACH1/MMP-9 axis that is effectively disrupted by TQ. Conclusions: This represents the first evidence that TQ exerts its anti-cancer effects in LC through the modulation of the critical signaling cascade (hsa-miR-155-5p → BACH1 → MMP-9). Our findings establish TQ as a multi-targeted agent capable of simultaneously inhibiting miRNA-mediated oncogenic signaling and protein-level effectors. The dual therapeutic action of TQ represents a novel therapeutic strategy and underscores its potential for synergistic combination therapies.

## 1. Introduction

Lung cancer (LC) poses a critical global health concern due to its high incidence and significant mortality rates. Contributing factors such as smoking, environmental pollutants, genetic predisposition, and disparities in healthcare access play major roles in its prevalence [1,2]. Moreover, the COVID-19 pandemic has further underscored regional differences in LC outcomes [3]. Despite advancements in diagnosis and treatment, LC remains the leading cause of cancer-related deaths globally [1,2,3], emphasizing the urgent need for innovative therapeutic approaches. MicroRNAs (miRNAs), which are small non-coding RNA molecules, are increasingly recognized as crucial regulators of gene expression in cancer. They influence key cellular functions, including cell growth, programmed cell death, and metastatic behavior [4]. While several miRNAs have been implicated in various cancers, their specific contributions to LC remain underexplored. Computational studies have identified numerous miRNA targets associated with LC, but experimental validation has been limited [5]. Among these, hsa-miR-155-5p has emerged as a prominent oncogenic miRNA. Its overexpression has been linked to tumor development and progression, correlating strongly with clinical and pathological features [6,7]. Dysregulation of miR-155-5p influences the expression of critical genes involved in LC, highlighting its potential as a therapeutic target [8,9]. Matrix metalloproteinase-9 (MMP-9), an enzyme integral to extracellular matrix remodeling, plays a pivotal role in LC by promoting tumor invasion, angiogenesis, and metastasis [10]. MMP-9’s involvement in immune evasion further establishes it as a critical player in cancer pathology. Targeting MMP-9 has shown promising results in preclinical studies, reducing metastatic potential and enhancing therapeutic efficacy [11]. Similarly, the transcription factor BACH1 (BTB and CNC Homology 1) has been identified as a key contributor to LC progression [12,13]. BACH1 regulates processes such as epithelial-to-mesenchymal transition (EMT), oxidative stress responses, and metabolic shifts, including the Warburg effect, which enables tumor adaptation under hypoxic conditions [14]. BACH1 has also been implicated as an important regulator of EMT, a biological process closely associated with tumor invasion, metastatic progression, and therapeutic resistance [12,13,14]. Previous studies have demonstrated that increased BACH1 activity promotes EMT-associated changes through regulation of key molecular mediators, including reduced epithelial markers such as E-cadherin and increased mesenchymal markers such as N-cadherin, Vimentin, Snail, and Twist [12,13,14]. These findings highlight the broader oncogenic role of BACH1 in promoting aggressive cancer phenotypes. However, the regulatory mechanisms connecting miRNA-mediated control of BACH1 expression with downstream metastatic mediators such as MMP-9 remain incompletely understood in lung cancer. Given its regulation of genes like MMP-9, BACH1 represents a promising therapeutic target to mitigate metastasis and improve treatment responses [12,13,14].

Thymoquinone (TQ), a bioactive compound from *Nigella sativa* (black seeds), has garnered attention for its diverse therapeutic properties, including anti-inflammatory, antioxidant, and anti-cancer effects [15,16,17]. Although TQ’s anti-cancer potential has been documented, its precise mechanisms, particularly in miRNA regulation within LC, remain inadequately understood. We hypothesized that TQ exerts its anti-cancer effects in LC by downregulating oncogenic hsa-miR-155-5p, thereby inhibiting the BACH1/MMP-9 signaling axis. Using bioinformatics and experimental approaches, we identified miRNA targets within the 3′ untranslated region (UTR) of BACH1 mRNA. Our findings reveal that TQ suppresses BACH1 and MMP-9 expression through hsa-miR-155-5p modulation in IFN-γ-stimulated or anti-miR-155-transfected A549 and SHP-77 LC cells. These insights suggest TQ’s potential as a targeted therapeutic agent for LC, providing a basis for future translational studies.

## 2. Methods

### 2.1. Cell Culture and Treatments

The human lung carcinoma cell line A549 is derived from human lung adenocarcinoma and represents a non-small cell lung cancer (NSCLC) model. A549 cells originate from alveolar type II epithelial cells and are widely used to investigate lung cancer biology, including tumor-associated signaling pathways, inflammatory responses, and therapeutic interventions [18]. On the other hand, the SHP-77 cell line is derived from human small-cell lung carcinoma (SCLC) and represents a neuroendocrine subtype of lung cancer. SHP-77 cells exhibit characteristic features of SCLC and serve as a valuable model for studying molecular mechanisms associated with aggressive lung cancer phenotypes [19]. Both cell lines were obtained from the American Type Culture Collection (ATCC, Manassas, VA, USA) and maintained under recommended culture conditions. Human lung carcinoma cell line A549 (ATCC, Manassas, VA, USA; Cellosaurus accession CVCL_0023) and human lung carcinoma cell line SHP-77 (ATCC, Manassas, VA, USA; Cellosaurus accession CVCL_1693) were cultured in RPMI 1640 medium (Sigma-Aldrich, St. Louis, MO, USA) enriched with 10% fetal bovine serum (FBS; Sigma-Aldrich Chemie GmbH, Taufkirchen, Germany) was obtained from the American Type Culture Collection (Rockville, MD, USA) and cultured in RPMI 1640 medium supplemented with 10% fetal bovine serum (FBS) and 1% penicillin–streptomycin solution at 37 °C in a humidified atmosphere containing 5% CO_2_ as described previously [20]. LC cells between passages 5 and 18 were used for all experiments to ensure consistency and avoid phenotypic drift. LC cells were serum-starved overnight to achieve 70–80% confluence before treatments. For viability assays, LC cells were exposed to thymoquinone (TQ; Sigma-Aldrich) under varying conditions, and viability was assessed using the Cell Titer-Glo Luminescent Assay (Promega, Madison, WI, USA). Additional experiments included TQ pre-treatment (50–100 nM) for 2 h, followed by stimulation with interferon-gamma (IFN-γ; EMD Millipore, Burlington, MA, USA) at 100 ng/mL, while untreated cells served as negative controls as previously described [20].

### 2.2. MicroRNA Target Prediction and Dual Luciferase Assay

Potential miRNA binding sites within the 3′ untranslated region (UTR) of BACH1 mRNA (ENST00000286800.3) were identified using TargetScan (http://www.targetscan.org/—accessed on 11 December 2025) as previously described [21]. Cloning of wild-type (Wt) and mutant (MUT) BACH1 sequences into pmirGLO luciferase reporter vectors (Promega) was performed using site-directed mutagenesis kits (Agilent, Santa Clara, CA, USA). A549 cells were co-transfected with reporter constructs, anti-miR-155-5p (Ambion, Austin, TX, USA/Qiagen, Venlo, The Netherlands), or anti-miR negative controls, and luciferase activity was measured after 48 h using the Dual Luciferase Reporter Assay System (Promega).

### 2.3. MicroRNA Transfection and Analysis

Lung cancer cells were transfected with 50 nM of anti-miR-155-5p inhibitors or equivalent concentrations of negative control miRNAs using HiPerfect Transfection Reagent (Qiagen). Post-transfection (72 h), LC cells were treated with TQ or stimulated with IFN-γ for 30 min to 24 h, followed by mRNA and protein extraction for downstream analyses as described previously [22].

### 2.4. RNA Isolation, cDNA Synthesis, and qPCR

Total RNA, including miRNAs, was extracted using the mirVana miRNA Isolation Kit (Ambion) according to the manufacturer’s instructions. For cDNA synthesis, 1 µg of total RNA was used for each reaction. First-strand cDNA synthesis was performed using the Superscript First-Strand cDNA Synthesis Kit (Applied Biosystems, Waltham, MA, USA). Quantitative PCR (qPCR) was conducted using TaqMan assays on the StepOne Real-Time PCR System (Life Technologies, Waltham, MA, USA). GAPDH and RNU6B were used as endogenous controls. Relative expression levels of BACH1, MMP-9, and miRNAs were calculated using the ΔΔCT method [23].

### 2.5. BACH1 Protein Quantification by Cell-Based Direct Binding ELISA

A direct binding ELISAs was used to quantify BACH1 protein levels as described previously with some modifications [24,25,26]. Briefly, A549 and SHP-77 LC cells were fixed, blocked, and incubated with primary antibodies against BACH1 (Cell Signaling Technology, Danvers, MA, USA), followed by secondary HRP-conjugated antibodies. Bound antibodies were detected using an alkaline phosphatase conjugate and quantified at 410 nm using a microplate reader (Anthos Zenyth; Biochrom Ltd., Cambridge, UK)).

### 2.6. Western Blotting and Densitometry

Western blot analysis was performed to evaluate secreted MMP-9 levels in concentrated cell culture supernatants. The supernatants were concentrated 10-fold using Amicon Ultra-4 centrifugal filter units with a 10 kDa molecular weight cutoff (Millipore) prior to SDS-PAGE analysis. Equal volumes of concentrated conditioned supernatants were loaded for analysis to ensure consistency among samples. Secreted proteins, along with Precision Plus Protein™ Standards (10–250 kDa, Bio-Rad, Hercules, CA, USA), were separated by SDS-PAGE and subsequently transferred onto PVDF membranes. The membranes were probed with 1:1000 diluted primary anti-MMP-9 antibodies (cat. #sc-21733, Santa Cruz Biotechnology, Dallas, TX, USA) and immunoreactive proteins were visualized by using 1:5000 diluted HRP-linked secondary antibodies Anti-Human IgG (cat. #32935, Cell Signaling Technology) as previously described [27,28]. Protein bands were visualized, and densitometric analysis was performed using UNSCAN-IT software, Version 6.1 (Silk Scientific, Provo, UT, USA). The MMP-9 protein detected by the Santa Cruz Biotechnology antibody showed an expected molecular weight between 75 and 100 kDa, corresponding to the MMP-9 form (~92 kDa).

### 2.7. Human MMP-9 ELISA Assay

MMP-9 secretion levels in the supernatants of lung cancer cells before and after treatments were quantified using a Human MMP-9 ELISA Kit (Thermo Fisher Scientific, Waltham, MA, USA), according to the manufacturer’s instructions.

### 2.8. Statistical Analysis

All experiments were performed in at least three independent biological replicates (*n* ≥ 3). Data were analyzed using one-way ANOVA followed by Tukey’s post hoc test or two-way ANOVA with Bonferroni corrections in GraphPad Prism 5 (San Diego, CA, USA). Statistical significance was set at *p* < 0.05.

## 3. Results

### 3.1. Thymoquinone and Interferon-γ Effects on Lung Cancer Cell Viability

**Figure 1:**
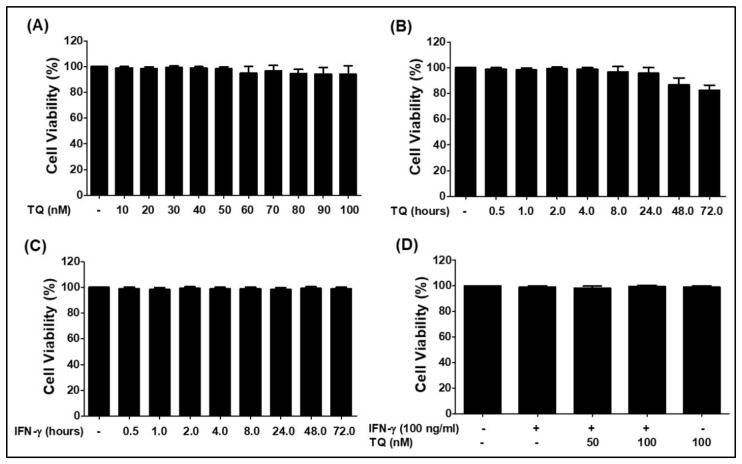
Influence of thymoquinone on lung cancer cell viability. (**A**) Viability of A549 LC cells (3 × 10^6^ cells/mL) in response to increasing concentrations of thymoquinone (TQ). LC cells were treated with TQ at concentrations ranging from 10 to 100 nM for 24 h. No significant difference was observed between control and TQ-treated cells (*p* > 0.05). (**B**) Viability of A549 cells (3 × 10^6^ cells/mL) over time following treatment with TQ. LC cells were exposed to 100 nM TQ for durations ranging from 0.5 to 72 h. No significant difference was observed between control and TQ-treated cells at different time points (*p* > 0.05). (**C**) Viability of A549 cells (3 × 10^6^ cells/mL) following varying durations of interferon-gamma (IFN-γ) treatment. LC cells were treated with 100 ng/mL IFN-γ for 0.5 to 72 h. No significant difference was observed between control and IFN-γ-treated cells (*p* > 0.05). (**D**) Viability of A549 cells (3 × 10^6^ cells/mL) after combined treatment with TQ and IFN-γ. LC cells were pretreated with 50–100 nM TQ for 0.5 h, followed by stimulation with 100 ng/mL IFN-γ for 24 h. No significant difference was observed between control and combined TQ/IFN-γ-treated cells (*p* > 0.05). All treatments were conducted in serum-starved RPMI medium at 37 °C with 5% CO_2_, and cell viability was assessed using the CellTiter-Glo Luminescent Assay (Promega, Madison, WI, USA). Data are presented as mean ± SD from three independent biological experiments (*n* = 3). Statistical analysis was performed using one-way ANOVA followed by Tukey’s post hoc test or two-way ANOVA with Bonferroni correction where appropriate.

The cytotoxic effects of TQ were assessed by treating A549 cells with concentrations ranging from 10 to 100 nM for 24 h, followed by viability analysis using the Cell Titer-Glo Luminescent Assay. The results indicated that TQ concentrations up to 100 nM did not significantly reduce cell viability compared to untreated controls (*p* > 0.05; Figure 1A). Further time-dependent studies revealed that treatment with 100 nM TQ for up to 72 h also maintained cell viability (Figure 1B). Similarly, IFN-γ treatment at 100 ng/mL for 0.5 to 72 h showed no significant cytotoxic effects on A549 cells (*p* > 0.05; Figure 1C). Combination treatments with 100 nM TQ and 100 ng/mL IFN-γ for 24 h demonstrated no additional toxicity (Figure 1D). Based on these findings, the selected concentrations for subsequent experiments were 100 nM TQ and 100 ng/mL IFN-γ.

### 3.2. Bioinformatics Prediction of hsa-miR-155-5p Binding to BACH1 mRNA

**Figure 2:**
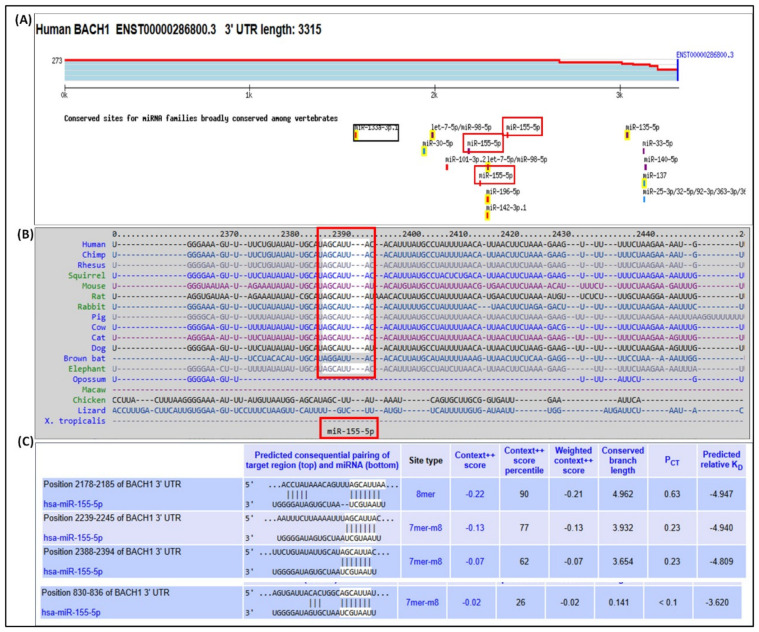
Bioinformatic analysis of the seed sequence of hsa-miR-155-5p within the 3′UTR of BACH1 mRNA. (**A**) Conserved binding sites of hsa-miR-155-5p (indicated in red rectangles) within the 3′UTR of human BACH1 mRNA (ENST00000286800.3). (**B**) TargetScan analysis showing conserved miR-155-5p sequences in the 3UTR of BACH1 mRNA across multiple species (highlighted in red rectangles). (**C**) Predicted duplex structure of hsa-miR-155-5p with its complementary sequence in the 3′UTR of BACH1 mRNA. Four conserved binding sites for hsa-miR-155-5p were identified, along with details of binding interactions, context scores, conservation metrics, and predicted relative Kd values.

TargetScan analysis identified four conserved binding sites for hsa-miR-155-5p within the 3′UTR of BACH1 mRNA (ENST00000286800.3). These sites were located at nucleotide positions 2178–2185, 2239–2245, 2388–2394, and 830–836 (Figure 2A,C). Comparative analysis showed conservation of these binding sites across multiple species, including humans, chimpanzees, mice, and pigs (Figure 2B). Context scores and predicted binding interactions further supported the potential for duplex formation between hsa-miR-155-5p and BACH1 mRNA.

### 3.3. Validation of Bioinformatics Predicted Interaction Between hsa-miR-155-5p and BACH1 mRNA 3′UTR

**Figure 3:**
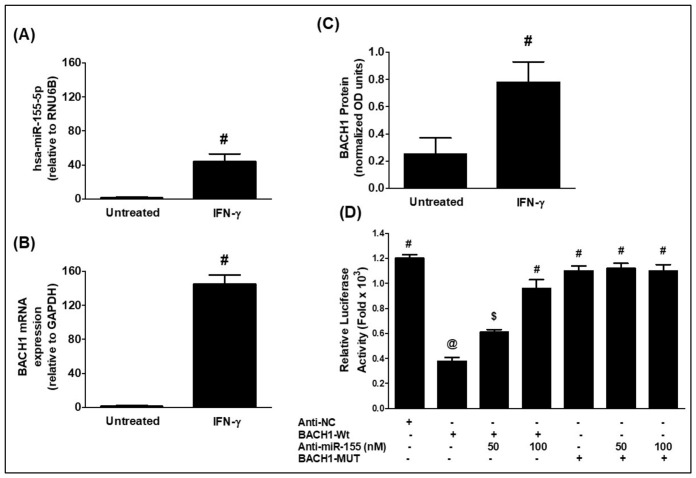
Relationship between hsa-miR-155-5p and BACH1 expression. (**A**) MicroRNA hsa-miR-155-5p expression in A549 cells (3 × 10^6^ cells/mL) treated with 100 ng/mL of IFN-γ for 3 h, measured using specific TaqMan assays. Untreated cells served as controls, with RNU6B used as an endogenous reference. Data represent mean ± SD from three independent experiments (# *p* < 0.01 vs. untreated cells). (**B**) BACH1 mRNA levels in A549 cells (3 × 10^6^ cells/mL) treated with 0.1 µg/mL of IFN-γ for 3 h, determined by BACH1-specific TaqMan assays. GAPDH served as an endogenous control. Data are shown as mean ± SD of three independent experiments (# *p* < 0.0001 vs. untreated cells). (**C**) BACH1 protein expression in A549 cells (3 × 10^6^ cells/mL) treated with 100 ng/mL of IFN-γ for 6 h, measured by cell-based binding ELISA. Data represent mean ± SD from three independent experiments (# *p* < 0.001 vs. untreated cells). (**D**) Luciferase activity in A549 cells transfected with BACH1-Wt (3′UTR reporter vector) and anti-miR-155-5p. Controls included anti-NC (negative control) and BACH1-MUT (mutant vector), with BACH1-Wt serving as a positive control. Data are presented as mean ± SD of three independent experiments (# *p* < 0.01 vs. @; $ *p* < 0.05 vs. #; @ *p* < 0.05 vs. $).

The interaction between hsa-miR-155-5p and BACH1 mRNA was examined in A549 lung cancer (LC) cells stimulated with IFN-γ. Upon treatment with 100 ng/mL IFN-γ for 3 h, a significant upregulation of hsa-miR-155-5p expression was observed (*p* < 0.05; Figure 3A). Concurrently, the stimulation markedly increased BACH1 expression at both the mRNA (*p* < 0.0001; Figure 3B) and protein levels (*p* < 0.01; Figure 3C). To further validate this interaction, dual luciferase reporter assays were performed by co-transfecting A549 cells with a BACH1-Wt reporter vector (containing the full-length 3′UTR of BACH1) and anti-miR-155. This co-transfection led to a dose-dependent rise in luciferase activity compared to cells transfected with BACH1-Wt alone (*p* < 0.05). Notably, transfection of BACH1-Wt alone resulted in reduced luciferase activity compared to cells transfected with BACH1-MUT or an anti-miR-negative control (*p* < 0.001). These findings confirm that hsa-miR-155-5p binds specifically to the conserved seed sequence within the 3′UTR of BACH1 mRNA (Figure 3D).

### 3.4. Thymoquinone Inhibits BACH1 mRNA and Protein Expression in IFN-γ-Stimulated Lung Cancer Cells

**Figure 4:**
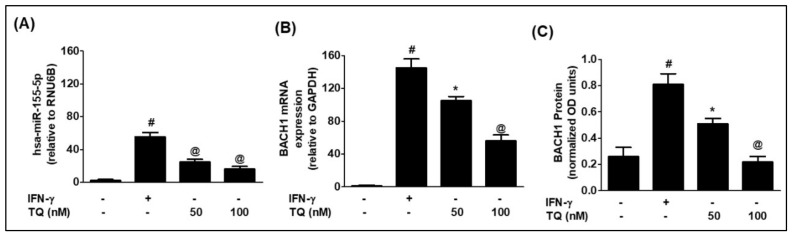
Regulation of hsa-miR-155-5p and BACH1 by thymoquinone in IFN-γ-treated lung cancer cells. (**A**) MicroRNA hsa-miR-155-5p expression determined using specific TaqMan assays. RNU6B served as an endogenous control. A549 cells (3 × 10^6^ cells/mL) were pretreated with 50–100 nM TQ for 2 h and then stimulated with IFN-γ for 3 h. Data are shown as mean ± SD of three experiments (# *p* < 0.001 vs. untreated cells; @ *p* < 0.05 vs. #). (**B**) BACH1 mRNA levels measured by TaqMan assays, using GAPDH as an endogenous reference. LC cells were treated as in (**A**). Data represent mean ± SD from three experiments (# *p* < 0.001 vs. untreated cells; * *p* < 0.05 vs. @; @ *p* < 0.01 vs. #). (**C**) BACH1 protein expression assessed via ELISA following similar treatments. Data represent mean ± SD from three experiments (* *p* <0.05 vs. @, # *p* < 0.01 vs. untreated cells; @ *p* < 0.01 vs. #).

The effect of thymoquinone (TQ) on IFN-γ-stimulated A549 LC cells was evaluated. Pre-treatment with TQ (50–100 nM) significantly suppressed the IFN-γ-induced upregulation of hsa-miR-155-5p in a dose-dependent manner (*p* < 0.05; Figure 4A). A similar reduction was observed in the expression of BACH1 mRNA in the same experimental conditions (*p* < 0.05; Figure 4B). To confirm these effects at the protein level, a cell-based ELISA was performed. TQ pre-treatment effectively reduced IFN-γ-stimulated BACH1 protein expression in a dose-dependent manner (*p* < 0.05; Figure 4C).

### 3.5. Thymoquinone Downregulates BACH1 Expression via hsa-miR-155-5p and the 3′UTR of BACH1 mRNA

**Figure 5:**
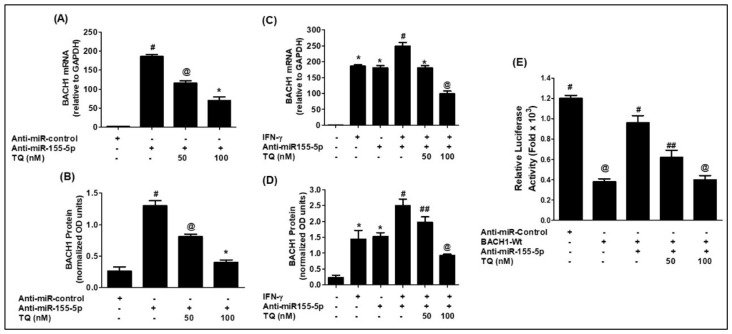
Thymoquinone reduces BACH1 expression by interacting with hsa-miR-155-5p in lung cancer cells. (**A**) Thymoquinone (TQ) suppresses BACH1 mRNA levels in A549 lung cancer cells transfected with anti-miR-155-5p. # *p* < 0.0001 compared to cells transfected with anti-miR-control; * *p* < 0.05 compared to @; @ *p* < 0.05 compared to #. (**B**) TQ decreases BACH1 protein expression in A549 cells transfected with anti-miR-155-5p. Statistical significance: # *p* < 0.001 compared to cells transfected with anti-miR-control; * *p* < 0.05 compared to @; @ *p* < 0.05 compared to #. (**C**) TQ inhibits IFN-γ-induced upregulation of BACH1 mRNA in A549 cells transfected with anti-miR-155-5p. # *p* < 0.0001; * *p* < 0.05; @ *p* < 0.05. (**D**) TQ reduces IFN-γ-induced BACH1 protein expression in A549 cells transfected with anti-miR-155-5p. * *p* < 0.01 compared to untreated cells; * *p* < 0.05 compared to #; # *p* < 0.05 compared to ##; # *p* < 0.001 compared to @; @ *p* > 0.05 compared to untreated cells. (**E**) TQ diminishes luciferase activity in A549 cells co-transfected with the BACH1-Wt (3′UTR reporter vector) and anti-miR-155-5p. Cells transfected with anti-miR-control alone were used as a negative control, while those transfected with BACH1-Wt alone served as a positive control. GAPDH expression was used as an endogenous reference. Data represent mean ± SD of three independent experiments. # *p* < 0.0001 compared to @; @ *p* < 0.05 compared to ##. Data are presented as mean ± SD from three independent biological experiments (*n* = 3).

The mechanism of TQ-induced inhibition of BACH1 expression was further examined using a combination of anti-miR-155-5p transfection and dual luciferase reporter assays. Transfection of A549 LC cells with anti-miR-155-5p significantly increased BACH1 mRNA levels compared to cells transfected with an anti-miR control (*p* < 0.001; Figure 5A). Interestingly, TQ treatment reversed this increase in a dose-dependent manner (*p* < 0.05). Similarly, the effect of TQ on BACH1 protein levels was evaluated, showing a significant reduction in protein expression in anti-miR-155-transfected cells treated with TQ (*p* < 0.05; Figure 5B). Co-treatment with TQ and IFN-γ further validated these findings, demonstrating that TQ inhibited IFN-γ-induced BACH1 mRNA and protein levels in anti-miR-155-transfected cells in a dose-dependent manner (*p* < 0.05; Figure 5C,D). Dual luciferase reporter assays confirmed that co-transfection with BACH1-Wt and anti-miR-155-5p increased luciferase activity (*p* < 0.01), which was significantly reduced upon TQ treatment in a dose-dependent fashion. These results suggest that TQ inhibits both hsa-miR-155-5p and BACH1 expression (Figure 5E).

### 3.6. TQ and BACH1 Inhibitor Suppress MMP-9 Expression Through hsa-miR-155-5p

**Figure 6:**
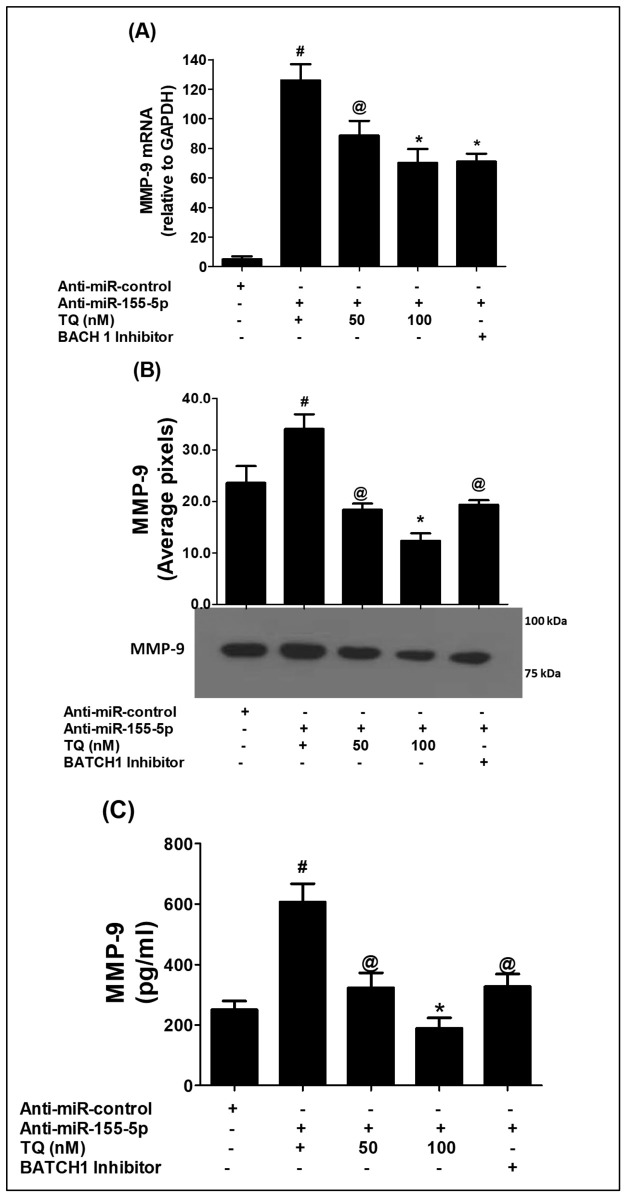
Thymoquinone and BACH1 inhibitor reduce MMP-9 expression in lung cancer cells transfected with anti-miR-155. (**A**) Thymoquinone (TQ) or the BACH1 inhibitor (ASP8731) downregulates MMP-9 mRNA levels in A549 lung cancer cells transfected with anti-miR-155, as measured by real-time PCR. GAPDH expression was used as an internal reference. Cells transfected with anti-miR-control served as the negative control, while those transfected with anti-miR-155-5p alone acted as the positive control. Data are presented as mean ± SD of three independent experiments. # *p* < 0.0001 compared to cells transfected with anti-miR-control; @ *p* < 0.05 compared to #; @ *p* < 0.05 compared to *. (**B**) TQ or ASP8731 reduces MMP-9 protein secretion in the culture medium of A549 cells transfected with anti-miR-155, as determined by Western blot analysis. The MMP-9 band was detected in between protein molecular weight standards 100 kDa and 75 kDa. Band intensities were digitally captured using Un-Scan-It software. Data represent mean ± SD of three independent experiments. Statistical significance: # *p* < 0.05 compared to cells transfected with anti-miR-control; @ *p* < 0.05 compared to #; @ *p* < 0.05 compared to *. (**C**) TQ or ASP8731 (BACH1 inhibitor) reduces MMP-9 protein secretion in the culture medium of A549 cells transfected with anti-miR-155, as determined by MMP-9 Sandwich ELISA Kit (Thermo Fisher Scientific). # *p* < 0.05 compared to cells transfected with anti-miR-control; @ *p* < 0.05 compared to #; @ *p* < 0.05 compared to *. Data represent mean ± SD of three independent experiments in all observations. The original WB images are shown in Figure S1.

The role of TQ and BACH1 in regulating MMP-9 expression was evaluated in A549 LC cells transfected with anti-miR-155-5p and treated with the BACH1 inhibitor ASP8731. Transfection with anti-miR-155-5p led to a significant increase in MMP-9 mRNA expression compared to anti-miR control-transfected cells (*p* < 0.0001; Figure 6A). This increase was significantly inhibited by TQ treatment in a dose-dependent manner (*p* < 0.05). The inhibitory effect of TQ on MMP-9 protein secretion was evaluated using complementary approaches. Western blot analysis demonstrated a reduction in secreted MMP-9 protein levels in conditioned culture medium following TQ treatment (*p* < 0.05; Figure 6B). These findings were further confirmed using a quantitative MMP-9-specific sandwich ELISA assay, which showed consistent suppression of MMP-9 secretion (*p* < 0.05; Figure 6C). Co-treatment with ASP8731 (BACH1 inhibitor) also suppressed MMP-9 mRNA and protein levels, confirming the involvement of BACH1 in this regulatory pathway (*p* < 0.05; Figure 6A,B and Figure S1). The agreement between Western blot and ELISA results strengthens the evidence that TQ regulates MMP-9 expression through modulation of the hsa-miR-155-5p/BACH1 signaling pathway in lung cancer cells.

### 3.7. Validation of Data Using an Additional Human Lung Cancer Cell Line (SHP-77)

**Figure 7:**
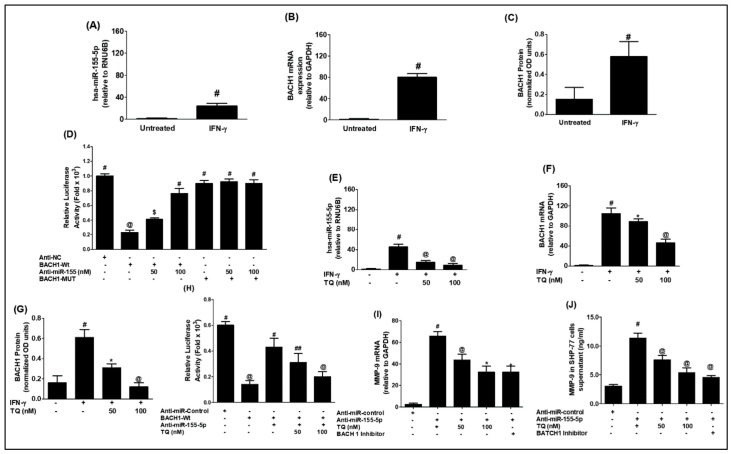
Thymoquinone reduces BACH1 expression and MMP-9 expression via interacting with hsa-miR-155-5p in SHP-77 human lung cancer cells. (**A**) MicroRNA hsa-miR-155-5p expression in SHP-77 cells (3 × 10^6^ cells/mL) treated with 100 ng/mL of IFN-γ for 3 h, measured using specific TaqMan assays. Untreated SHP-77 cells served as controls, with RNU6B used as an endogenous reference. # *p* < 0.05 vs. untreated SHP-77 cells. (**B**) BACH1 mRNA levels in SHP-77 cells treated with 0.1 µg/mL of IFN-γ for 3 h, determined by BACH1-specific TaqMan assays. GAPDH served as an endogenous control. # *p* < 0.001 vs. untreated cells. (**C**) BACH1 protein expression in SHP-77 cells treated with 100 ng/mL of IFN-γ for 6 h, measured by cell-based binding ELISA. # *p* < 0.05 vs. untreated SHP-77 cells. (**D**) Luciferase activity in SHP-77 cells transfected with BACH1-Wt (3′UTR reporter vector) and anti-miR-155-5p. Controls included anti-NC (negative control) and BACH1-MUT (mutant vector), with BACH1-Wt serving as a positive control. # *p* < 0.05 vs. @; $ *p* < 0.05 vs. #; @ *p* < 0.05 vs. $. (**E**) MicroRNA-155-5p expression determined using specific TaqMan assays. RNU6B served as an endogenous control. SHP-77 cells were pretreated with 50-100 nM TQ for 2 h and then stimulated with IFN-γ for 3 h. # *p* < 0.001 vs. untreated SHP-77 cells; @ *p* < 0.05 vs. #. (**F**) BACH1 mRNA levels measured by TaqMan assays, using GAPDH as an endogenous reference. # *p* < 0.001 vs. untreated SHP-77 cells; * *p* < 0.05 vs. @; @ *p* < 0.01 vs. #. (**G**) BACH1 protein expression assessed via ELISA following similar treatments. # *p* < 0.01 vs. untreated SHP-77 cells; @ *p* < 0.01 vs. #. * *p* < 0.05. (**H**) TQ reduces luciferase activity in SHP-77 cells co-transfected with the BACH1-Wt and anti-miR-155-5p. SHP-77 cells transfected with anti-miR-control alone were used as a negative control, while those transfected with BACH1-Wt alone served as a positive control. GAPDH expression was used as an endogenous reference. # *p* < 0.0001 compared to @; @ *p* < 0.05 compared to ##. (**I**) TQ or the BACH1 inhibitor (ASP8731) downregulates MMP-9 mRNA levels in SHP-77 cells transfected with anti-miR-155. # *p* < 0.0001 compared to SHP-77 cells transfected with anti-miR-control; @ *p* < 0.05 compared to #; @ *p* < 0.05 compared to *. (**J**) TQ or ASP8731 reduces MMP-9 protein secretion in the culture medium of SHP-77 cells transfected with anti-miR-155, as determined by MMP-9 Sandwich ELISA Kit (Thermo Fisher Scientific). # *p* < 0.05 compared to cells transfected with anti-miR-control; @ *p* < 0.05 compared to #; @ *p* < 0.05 compared to *. Data are presented as mean ± SD from three independent biological experiments (*n* = 3). Statistical analysis was performed using one-way ANOVA followed by Tukey’s post hoc test or two-way ANOVA with Bonferroni correction where appropriate.

**Figure 8:**
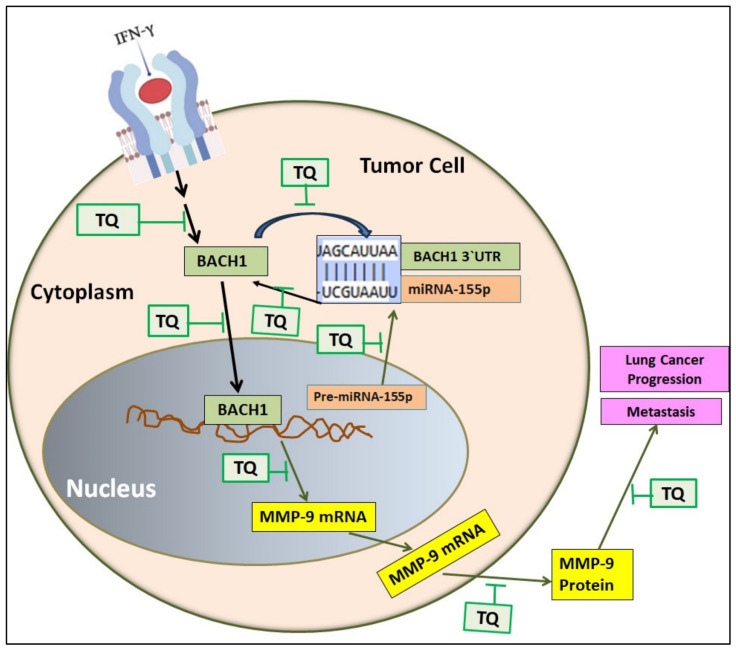
Overview of thymoquinone-mediated therapeutic targets. Schematic representation of TQ’s effects on IFN-γ-activated BACH1 signaling, miR-155-5p regulation, and subsequent suppression of MMP-9 expression in lung cancer cells, highlighting its potential to reduce metastasis and progression.

To further validate the therapeutic role of TQ, experiments were performed using an additional human lung cancer cell line, SHP-77. The cells were pretreated with TQ (50–100 nM) prior to IFN-γ exposure or transfected with anti-miR-155. The findings obtained from SHP-77 cells were comparable to those observed in A549 lung cancer cells. The complete data generated using SHP-77 cells are presented in Figure 7. Collectively, the consistent results obtained from both A549 and SHP-77 lung cancer cell lines suggest that inhibition of inflammatory pathways plays a crucial role in the regulatory effects of TQ. Together, these results demonstrate that TQ inhibits MMP-9 expression by modulating hsa-miR-155-5p and BACH1 (Figure 7). The overall effects of TQ on IFN-γ-activated BACH1 signaling, hsa-miR-155-5p regulation, and MMP-9 suppression, along with its potential to limit LC progression and metastasis, are summarized in Figure 8.

## 4. Discussion

This is the first study that highlights the potential therapeutic role of thymoquinone in modulating oncogenic pathways in lung cancer, specifically via the regulation of hsa-miR-155-5p, BACH1, and MMP-9. Our results demonstrate that TQ can effectively inhibit IFN-γ-induced expression of BACH1 and MMP-9, pivotal players in lung cancer progression and metastasis, through the modulation of hsa-miR-155-5p expression. These findings add to the growing evidence supporting the role of miRNA-targeted therapeutics in cancer treatment. BACH1 (BTB and CNC homology 1) has been identified as a critical transcription factor in cancer biology, particularly in LC [13,29]. It plays a significant role in promoting epithelial-to-mesenchymal transition (EMT), a process essential for tumor invasion and metastasis [30]. BACH1’s involvement in oxidative stress regulation and metabolic reprogramming, including the Warburg effect, has been well-documented [31]. These functions enable tumor survival under hypoxic conditions and enhance metastatic potential through upregulation of genes like MMP-9 [12,13,14,31]. Furthermore, elevated BACH1 levels have been associated with poor prognosis and resistance to therapy in LC patients. Recent studies suggest that BACH1 also plays a role in regulating ferroptosis, a type of programmed cell death linked to iron metabolism and lipid peroxidation, further emphasizing its multifaceted role in cancer pathophysiology [32,33]. Our results demonstrated that TQ significantly downregulated BACH1 expression at both mRNA and protein levels, indicating its potential to suppress these oncogenic pathways and reduce tumor aggressiveness.

Matrix metalloproteinase-9 (MMP-9) is a proteolytic enzyme crucial for extracellular matrix degradation, facilitating tumor invasion and angiogenesis. Its expression has been linked to advanced stages of LC and poor clinical outcomes [10,11]. MMP-9 also contributes to immune evasion, further underscoring its role in cancer progression [34,35,36]. It is regulated by multiple signaling pathways, including NF-κB and MAPK, which are activated in response to cytokines and growth factors [10,11,34]. Our study found that TQ treatment effectively reduced MMP-9 expression, even in the presence of IFN-γ stimulation or anti-miR-155-5p transfection, highlighting the interplay between BACH1 and MMP-9 in TQ’s anti-metastatic effects. Moreover, the synergistic inhibition of MMP-9 expression by TQ and the BACH1 inhibitor ASP8731 provides compelling evidence of a regulatory axis involving hsa-miR-155-5p, BACH1, and MMP-9. These findings align with previous reports that targeting MMP-9 can enhance the efficacy of anti-tumor therapies [35,36], suggesting that TQ may have utility as part of a combinatorial treatment strategy.

MicroRNAs are increasingly recognized as pivotal regulators in cancer biology, influencing processes such as proliferation, apoptosis, and metastasis. Among these, hsa-miR-155-5p has emerged as an oncogenic miRNA with significant roles in LC [37,38]. Its overexpression has been reported in LC tissues and correlates with aggressive tumor behavior, including increased proliferation, invasion, and resistance to apoptosis [37]. By directly targeting the 3′UTR of BACH1 mRNA, hsa-miR-155-5p promotes BACH1’s oncogenic functions, creating a feedback loop that drives cancer progression. Moreover, miR-155 has been implicated in immune modulation, influencing the tumor microenvironment by altering cytokine production and immune cell infiltration [37,38].

Previous studies have demonstrated that IFN-γ can induce miR-155 expression through inflammatory signaling pathways, supporting its use as a model to investigate miR-155-associated regulatory mechanisms [39,40]. Although miRNAs typically function as negative regulators of their target genes, the regulation of gene expression within inflammatory tumor microenvironments involves complex interactions between multiple signaling pathways. In the present study, IFN-γ stimulation increased both hsa-miR-155-5p and BACH1 expression in lung cancer cells. This simultaneous induction suggests that IFN-γ-mediated inflammatory signaling may directly activate BACH1 expression through upstream transcriptional mechanisms, while increased hsa-miR-155-5p expression may act as a compensatory feedback mechanism to regulate excessive BACH1 activation. Therefore, the increase in miR-155-5p following IFN-γ stimulation does not necessarily indicate complete suppression of BACH1, particularly under strong cytokine-driven activation conditions. To further clarify this regulatory relationship, we used anti-miR-155-5p-mediated inhibition and BACH1 3′UTR luciferase reporter assays. Suppression of endogenous hsa-miR-155-5p resulted in increased BACH1 mRNA and protein expression, confirming the direct regulatory interaction between miR-155-5p and BACH1. Furthermore, enhanced BACH1 expression following miR-155-5p inhibition was associated with increased MMP-9 expression, supporting the role of BACH1 as an upstream regulator of MMP-9-mediated metastatic signaling. These findings suggest that hsa-miR-155-5p functions as an important regulatory component within the IFN-γ-induced BACH1/MMP-9 signaling pathway rather than acting as the sole determinant of BACH1 expression. Importantly, TQ treatment effectively suppressed IFN-γ-induced activation of this signaling cascade by reducing hsa-miR-155-5p-associated dysregulation, BACH1 expression, and downstream MMP-9 production. Thus, our findings identify a novel mechanism by which TQ modulates the inflammatory hsa-miR-155-5p/BACH1/MMP-9 axis in lung cancer cells.

Recent studies have highlighted its ability to modulate key signaling pathways such as PI3K/Akt, JAK/STAT, and NF-κB, which are crucial in cancer development and progression [15,17]. In LC, TQ has been shown to induce apoptosis, inhibit angiogenesis, and sensitize tumors to chemotherapeutic agents [41]. Our results expand on these findings by demonstrating that TQ also modulates miRNA expression, specifically targeting hsa-miR-155-5p, BACH1, and MMP-9. This dual mechanism of action of direct inhibition of oncogenic signaling and miRNA modulation highlights the therapeutic versatility of TQ. Furthermore, the interplay between BACH1 and MMP-9 highlights a broader implication for targeted therapies, wherein the BACH1 transcriptionally mediated activation of MMP-9 is crucial for tumor metastasis. By inhibiting this pathway, TQ not only impairs tumor invasion but also enhances sensitivity to chemotherapy and immunotherapy. This dual mechanism of action positions TQ as a promising candidate for combination therapy in LC treatment. Moreover, our study provides insights into the potential of TQ as an adjuvant to existing therapies. By targeting multiple components of the oncogenic signaling network including transcription factors, proteolytic enzymes, and miRNAs, TQ offers a multi-faceted approach to combat LC progression. Importantly, the inhibitory effects of TQ on hsa-miR-155-5p, BACH1, and MMP-9 expression were consistently observed in both A549 lung adenocarcinoma cells and SHP-77 small-cell lung carcinoma cells, demonstrating that the anti-oncogenic effects of TQ are not restricted to a single lung cancer subtype. In both cellular models, TQ effectively suppressed IFN-γ-induced inflammatory signaling and reduced the expression of key mediators involved in tumor progression and metastasis. These findings strengthen the biological significance and reproducibility of the proposed hsa-miR-155-5p/BACH1/MMP-9 regulatory axis. Several previous studies have established the individual roles of miR-155, BACH1, and MMP-9 in cancer progression. Increased miR-155 expression has been reported in multiple malignancies, including lung, breast, colorectal, and hematological cancers, where it contributes to tumor growth, inflammatory signaling, invasion, and therapeutic resistance [37,38,39]. Similarly, BACH1 has emerged as an important transcriptional regulator associated with epithelial-to-mesenchymal transition (EMT), oxidative stress adaptation, metabolic reprogramming, and enhanced metastatic potential in different tumor types [14]. Previous reports have also demonstrated that BACH1 can regulate metastasis-associated genes, including MMP-9, thereby promoting extracellular matrix remodeling and cancer cell invasion. Consistent with these observations, our findings demonstrate that activation of the miR-155-5p/BACH1 pathway contributes to increased MMP-9 expression in lung cancer cells. Elevated BACH1 expression has been associated with EMT activation through modulation of EMT-related markers, including suppression of E-cadherin and induction of mesenchymal regulators such as N-cadherin, Vimentin, Snail, and Twist [14]. Although EMT markers were not directly examined in the present study, our findings demonstrate that TQ effectively suppresses BACH1 expression and its downstream metastasis-related target MMP-9 through regulation of the hsa-miR-155-5p/BACH1 signaling pathway. These observations are consistent with previous reports linking BACH1 activation with enhanced metastatic potential. Future studies investigating the effect of TQ on EMT-specific markers will further clarify whether inhibition of the miR-155-5p/BACH1/MMP-9 axis also contributes to EMT suppression in lung cancer.

TQ, the major bioactive component of *Nigella sativa*, has been widely reported to exert anticancer effects through modulation of inflammatory pathways, oxidative stress, apoptosis, and metastasis-associated signaling in various cancers. Previous studies have shown that TQ can suppress tumor progression through regulation of key pathways such as NF-κB, STAT3, PI3K/Akt, and MAPK signaling [15,17,40]. However, limited information is available regarding the ability of TQ to regulate miRNA-mediated oncogenic pathways in lung cancer. In contrast to previous studies mainly focusing on conventional signaling pathways, the present study identifies a novel mechanism whereby TQ suppresses the hsa-miR-155-5p/BACH1/MMP-9 signaling axis, resulting in reduced expression of the metastasis-associated mediator MMP-9. Therefore, while previous studies have independently described the oncogenic functions of miR-155, BACH1, and MMP-9 [6,7,8,9,10,12,37,38,39], our findings provide new evidence linking these molecules within a single regulatory cascade in lung cancer. The ability of TQ to target this interconnected miR-155-5p/BACH1/MMP-9 pathway represents a novel mechanistic insight and further supports the therapeutic potential of TQ as a multi-target anticancer agent for controlling lung cancer progression and metastasis.

These findings have been validated in two established human lung cancer cell lines, A549 and SHP-77, which are widely used models for lung cancer research and therapeutic investigations [42,43]. Future studies incorporating in vivo animal models and gene knockout approaches will be essential to further validate and expand upon these findings and to support the translational and clinical potential of TQ as a therapeutic strategy for lung cancer. One limitation of the present study is that the effects of TQ were evaluated only in lung cancer cell models, while normal lung epithelial cells were not included. Assessment of TQ responses in normal lung cells would provide additional insights into its cancer-selective activity, potential cytotoxicity, and overall safety profile. Although our findings demonstrate the regulatory effect of TQ on the hsa-miR-155-5p/BACH1/MMP-9 signaling axis in lung cancer cells, future studies comparing malignant and non-malignant lung cells are required to further validate the therapeutic selectivity and translational potential of TQ. Another limitation of this study is that complete rescue experiments involving miR-155-5p overexpression and BACH1 overexpression were not performed. Although anti-miR-155-5p inhibition, BACH1 3′UTR luciferase reporter assays, and BACH1 inhibitor experiments provided mechanistic evidence supporting the involvement of the miR-155-5p/BACH1/MMP-9 pathway, future gain-of-function studies are required to further confirm whether restoration of miR-155-5p or BACH1 expression can directly reverse the effects of TQ on this signaling axis. In short, our results suggest that TQ exerts anti-oncogenic effects in LC by modulating hsa-miR-155-5p and its downstream targets, BACH1 and MMP-9. This novel mechanism of action highlights TQ’s potential as a therapeutic agent for inhibiting LC progression and metastasis. Future studies should aim to explore its clinical utility and integration into standard treatment protocols.

## 5. Conclusions

The present study demonstrates that TQ modulates the hsa-miR-155-5p/BACH1/MMP-9 signaling axis in lung cancer (LC) cell models, providing new insights into a potential molecular mechanism underlying its anticancer effects by regulating inflammatory and metastasis-associated pathways, including suppression of BACH1 and downstream MMP-9 expression at the mRNA and protein levels; however, as these findings are based on in vitro experimental models, further validation using in vivo studies and clinical samples is required to confirm the physiological relevance, therapeutic applicability, and potential incorporation of TQ into future lung cancer treatment strategies.

## Data Availability

All data and materials used in this study are available within study.

## Supplementary Materials

The following supporting information can be downloaded at https://www.mdpi.com/article/10.3390/biom16070955/s1, Figure S1: Original Western Blot.
