# Supplementary figures and images for: Targeting the hsa-miR-155-5p–BACH1–MMP-9 Signaling Hub in Lung Cancer: A Novel Anticancer Mechanism of Thymoquinone
Source: Biomolecules. 2026 Jun 27;16(7):955. doi: 10.3390/biom16070955 (PMC13406729; doi:10.3390/biom16070955)

Figure S1: Original Western Blot\_MMP9

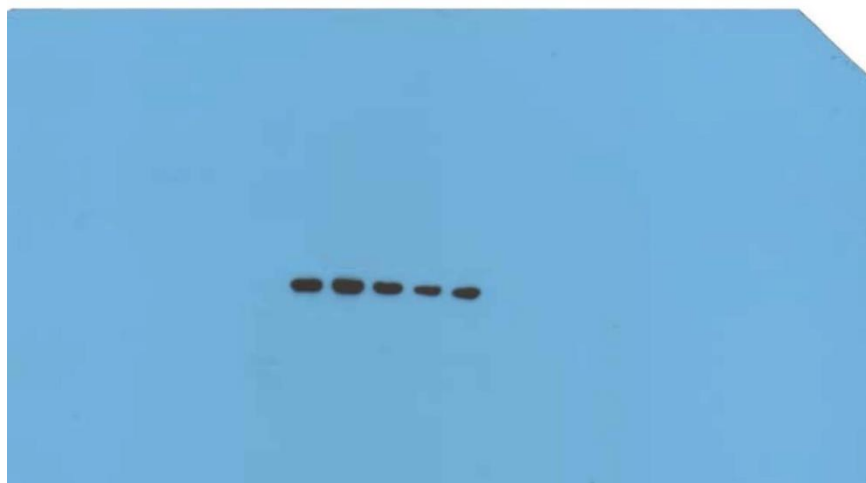

Supplement: Supplementary file 1 [file biomolecules-16-00955-s001.zip › biomolecules-4343614-supplementary.pdf]
